# Different regulation of cigarette smoke induced inflammation in upper versus lower airways

**DOI:** 10.1186/1465-9921-11-100

**Published:** 2010-07-23

**Authors:** Wouter Huvenne, Claudina A Pérez-Novo, Lara Derycke, Natalie De Ruyck, Olga Krysko, Tania Maes, Nele Pauwels, Lander Robays, Ken R Bracke, Guy Joos, Guy Brusselle, Claus Bachert

**Affiliations:** 1 Upper Airways Research Laboratory (URL), ENT Department, Ghent University Hospital, Ghent University, Belgium; 2 Department of Respiratory Medicine, Ghent University Hospital and Ghent University, Ghent, Belgium

## Abstract

**Background:**

Cigarette smoke (CS) is known to initiate a cascade of mediator release and accumulation of immune and inflammatory cells in the lower airways. We investigated and compared the effects of CS on upper and lower airways, in a mouse model of subacute and chronic CS exposure.

**Methods:**

C57BL/6 mice were whole-body exposed to mainstream CS or air, for 2, 4 and 24 weeks. Bronchoalveolar lavage fluid (BAL) was obtained and tissue cryosections from nasal turbinates were stained for neutrophils and T cells. Furthermore, we evaluated GCP-2, KC, MCP-1, MIP-3α, RORc, IL-17, FoxP3, and TGF-β1 in nasal turbinates and lungs by RT-PCR.

**Results:**

In both upper and lower airways, subacute CS-exposure induced the expression of GCP-2, MCP-1, MIP-3α and resulted in a neutrophilic influx. However, after chronic CS-exposure, there was a significant downregulation of inflammation in the upper airways, while on the contrary, lower airway inflammation remained present. Whereas nasal FoxP3 mRNA levels already increased after 2 weeks, lung FoxP3 mRNA increased only after 4 weeks, suggesting that mechanisms to suppress inflammation occur earlier and are more efficient in nose than in lungs.

**Conclusions:**

Altogether, these data demonstrate that CS induced inflammation may be differently regulated in the upper versus lower airways in mice. Furthermore, these data may help to identify new therapeutic targets in this disease model.

## Background

Tobacco smoking can induce bronchial inflammation and structural changes, and is one of the major causes of Chronic Obstructive Pulmonary Disease (COPD), which is characterized by a slowly progressive development of airflow limitation that is not fully reversible [1]. There is growing evidence that the disease process is not confined to the lower airways, which is perhaps not surprising given the fact that the entire airway is exposed to tobacco smoke. Epidemiological data suggest that 75% of the COPD patients have concomitant nasal symptoms and more than 1/3 of patients with sinusitis also have lower airway symptoms of asthma or COPD [2]. These arguments stress the significant sinonasal inflammation in patients with lower airway complaints, beyond the scope of allergic inflammation [3-5].

We know from human and murine research that both inflammatory and structural cells actively participate in the inflammatory response that characterizes COPD. An accumulation of inflammatory cells such as neutrophils, macrophages, dendritic cells and CD8+ T lymphocytes is seen, although the cellular and molecular pathways behind this increased cellular influx are still incompletely unraveled. However, CC-chemokines (MIP-1alpha, MIP-3alpha, RANTES and MCP-1) [6] and CXC-chemokines (IL-8, GCP-2) [7], binding to their respective receptors play an important role. Moreover, the role of lymphocytes in the development of COPD is demonstrated by the fact that chronic cigarette smoke (CS) exposure leads to an increase in peribronchial lymphoid follicles in both mice and humans [8,9], although the importance of these lymphoid follicles remains unclear [10].

COPD is frequently considered a Th1/Tc1 disease [11], although recent developments in cytokine biology imply that COPD might be better explained by the pro-inflammatory T helper 17 (Th17) phenotype [12], therefore suggesting a role of the interleukin (IL)-17 family members in COPD [13]. Alternatively, T regulatory cells which are widely investigated in the pathogenesis of asthma, might be involved in a possible autoimmune base of COPD [14]. These cells, expressing the transcription factor FoxP3, are involved in the interplay between lymphocyte subpopulations in order to control the cigarette smoke induced inflammation, including the activity of autoreactive lymphocytes [15].

Compared to lungs, the direct effect of CS on upper airways is less extensively studied, although the link between upper and lower airway smoke induced inflammation is illustrated by increased nasal IL-8 concentrations correlating with IL-8 in sputum of COPD patients [2]. Moreover, these patients report a high prevalence of nasal symptoms and sinusitis, and nasal and bronchial inflammation coexist in smokers and is characterized by infiltration of CD8+ T lymphocytes [16]. In upper airways, CS may act as a local irritant, influencing the local inflammatory process. It has been described that nicotine has an effect on the nasal epithelium, regulating physiological processes and influencing cell transport systems [17], although an individual variability in response has been reported. CS can increase nasal resistance [18], and the direct use of tobacco could also be linked to an increased prevalence of sinusitis [19]. In addition, a correlation between duration of secondhand smoke exposure and sinusitis has recently been described [20].

Also in mice, obligatory nose breathers, little knowledge has been gathered on the effects of CS on upper airways, especially in comparison to the lower airways. We therefore aimed to investigate the inflammatory response of the upper airways in a murine model of COPD in comparison to the lower airway response after exposure to mainstream cigarette smoke.

## Methods

### Mouse model of Cigarette Smoke exposure

Groups of 8 Male C57BL/6 mice, 6-8-week old were exposed to the tobacco smoke of five cigarettes (Reference Cigarette 2R4F without filter; University of Kentucky, Lexington, KY, USA) four times per day with 30 min smoke-free intervals as described previously [6]. The animals were exposed to mainstream cigarette smoke by whole body exposure, 5 days per week for 2 weeks, 4 weeks and 24 weeks. The control groups (8 age-matched male C57BL/6 mice) were exposed to air. All experimental procedures were approved by the local ethical committee for animal experiments (Faculty of Medicine and Health Sciences, Ghent University).

### Bronchoalveolar lavage

Twenty-four hours after the last exposure, mice were weighed and sacrificed with an overdose of pentobarbital (Sanofi-Synthelabo), and a tracheal cannula was inserted. A total of 3 × 300 μl, followed by 3 × 1 ml of HBSS, free of ionized calcium and magnesium, but supplemented with 0.05 mM sodium EDTA, was instilled via the tracheal cannula and recovered by gentle manual aspiration. The six lavage fractions were pooled and centrifuged, and the cell pellet was washed twice and finally resuspended in 1 ml of HBSS. A total cell count was performed in a Bürcker chamber, and the differential cell counts (on at least 400 cells) were performed on cytocentrifuged preparations using standard morphologic criteria after May-Grünwald-Giemsa staining.

### Quantitative real time PCR

#### RNA and cDNA synthesis

Total RNA was isolated from mouse inferior turbinate or lung tissue by using the Aurum Total RNA Mini Kit (BioRad Laboratories, CA, USA). Single stranded cDNA was then synthesized from 2 μg of total RNA with the iScript cDNA Synthesis Kit (BioRad Laboratories, CA, USA). Primer sequences are listed in table 1.

**Table 1:** Primer sequences used for real time PCR amplification

	**Forward primer (5'→ 3')**	**Reverse primer (5'→ 3')**	**TaqMan probe (5'-6-FAM → TAMRA-3')**	**Amplicon size**	**Genbank Accession number**
ACTB	AGAGGGAAATCGTGCGTGAC	CAATAGTGATGACCTGGCCGT	CACTGCCGCATCCTCTTCCTCCC	139	NM_007393
GCP-2	GCTGCCCCTTCCTCAGTCAT	CACCGTAGGGCACTGTGGA		129	NM_009141
MCP-1	CTTCTGGGCCTGCTGTTCA	CCAGCCTACTCATTGGGATCA	CTCAGCCAGATGCAGTTAACGCCCC	126	NM_011 333
MIP-3α	CCAGGCAGAAGCAAGCAACT	TCGGCCATCTGTCTTGTGAA	TGTTGCCTCTCGTACATACAGACGCCA	71	AJ222694 1
TGF-β1	TGACGTCACTGGAGTTGTACGG	GGTTCATGTCATGGATGGTGC	TTCAGCGCTCACTGCTCTTGTGACAG	170	M13177

RORc: Applied Biosystems - TaqMan Gene Expression Assays - Mm00441139_m1

KC (CXCL1): Applied Biosystems - TaqMan Gene Expression Assays - Mm00433859_m1

FoxP3: Applied Biosystems - TaqMan Gene Expression Assays - Mm00475156_m1

IL-17: Applied Biosystems - TaqMan Gene Expression Assays - Mm00439619_m1

#### PCR amplifications using SYBR Green

PCR reactions contained 30 ng cDNA (total RNA equivalent) of each sample in duplicate, 1× SYBR Green I Master mix (BioRad laboratories, CA, USA) and 250 nM of specific primer pairs (table 1) in a final volume of 20 μl. Real time amplifications were performed on the iQ5 Real-Time PCR Detection System (BioRad laboratories, CA, USA) with a protocol consisting of 1 cycle at 95°C for 10 minutes followed by 40 cycles at 95°C for 30 seconds and at 62°C for 1 minute. At the end of each PCR run, a melting curve analysis to control for unspecific amplification was performed by increasing the temperature by 0.4°C for 10 seconds starting from 62°C until 95°C.

#### PCR amplifications using TaqMan probes

PCR reactions contained 30 ng cDNA (total RNA equivalent) of each sample in duplicate, 1× TaqMan Master mix (BioRad laboratories, CA, USA), 100 nM of TaqMan probe and 250 nM of specific primer pairs (table 1) in a final volume of 20 μl. Real time amplifications were performed on the iQ5 Real-Time PCR Detection System (BioRad laboratories, CA, USA) with a protocol consisting of 1 cycle at 95°C for 90 seconds followed by 50 cycles at 95°C for 15 seconds, 62°C for 1 minute and 72°C for 1 minute.

#### PCR amplifications using Assay on demand kits

PCR reactions contained 30 ng cDNA (total RNA equivalent) of each sample in duplicate and 1× TaqMan Master mix (BioRad laboratories, CA, USA). Primers were obtained from Applied Biosystems inventoried TaqMan Gene Expression Assay (table 1). Real time amplifications were performed on the iQ5 Real-Time PCR Detection System (BioRad laboratories, CA, USA) with a protocol consisting of 1 cycle at 95°C for 90 seconds followed by 50 cycles at 95°C for 15 seconds and 60°C for 1 minute.

#### Normalization and data analysis

Quantification cycles (Cq) values were selected and analyzed using the iQ5 Real-Time PCR software (BioRad laboratories, CA, USA). Then, the relative expression of each gene was calculated with the qBase software (version 1.3.5, University of Ghent, Belgium) [21]. Results (expressed as relative expression units/30 ng cDNA) were then normalized to the quantities of gene beta-actin (ACTB) to correct for transcription and amplification variations among samples.

### Immunohistochemistry

#### Presence of lymphoid follicles

To evaluate the presence of lymphoid infiltrates in lung tissues, sections obtained from formalin-fixed, paraffin-embedded lung lobes were subjected to an immunohistological CD3/B220 double-staining, as described previously [6].

#### Inferior turbinate stainings

After removal of the palate, nasal turbinates were obtained, snap frozen and stored at -80°C until analysis. Cryosections were prepared (3-5 μm) and mounted on SuperFrost Plus glass slides (Menzel Glaeser, Braunschweig, Germany), packed in aluminum paper and stored at -20°C until staining.

Sections were fixed in acetone and incubated with peroxidase blocking reagent. Then, primary biotinylated antibodies (anti-CD3 (DakoCytomation, CA, USA) and neutrophil 7/4 clone (Serotec, Düsseldorf, Germany)) or isotype control were added, followed by anti-rabbit polymer HRP (DakoCytomation). Finally, ready-to-use AEC+ substrate-chromogen-solution was added, sections were counterstained with hematoxylin and coverslips were mounted with aquatex. Slides were evaluated by light microscopy (Olympus CX40) at magnification of x400 for the number of positive cells per field, and this was done for the entire surface of the tissue cryosection by two independent observers (on average, 12.43 ± 1.00 number of fields were counted per mouse).

### Nasal epithelial cell isolation

Nasal epithelial cells were isolated in order to determine their contribution to the overall nasal FoxP3 expression. Therefore, pooled inferior turbinates were incubated in collagenase/DNAse solution for 30 min at 37°C. Then, mechanical digestion was performed, and supernatant was discarded. The pellet was washed and incubated for 30 min at 4°C with Fc blocking solution. Next, Dynabeads (sheep anti-mouse IgG, Dynal, Invitrogen, Belgium) coated with anti-pan cytokeratin (catalog nr C 1801, Sigma, Belgium) were for 30 min at 4°C during gentle rotation and tubes were placed in the magnet for 2 min. The two fractions containing epithelial and subepithelial cells respectively, were resuspended in 75 μl RNA lysis buffer (Qiagen, Venlo, The Netherlands) in separate tubes. Finally, tubes containing subepithelial cells were centrifuged, and tubes containing epithelial cells were put again in the magnet. Supernatant was taken to store at -80°C.

In order to isolate total RNA from nasal epithelial cells and subepithelial cells, we used the RNeasy Micro kit (Qiagen) according to the manufacturer's specifications. Single stranded cDNA was then synthesized from 2 μg of total RNA with the iScript cDNA Synthesis Kit (BioRad Laboratories).

### Statistical analysis

Statistical analysis was performed with the Medcalc software 9.2.0.1 (F. Schoonjans, Belgium, http://www.medcalc.be). Data are expressed as mean with error bars expressing standard error of the mean. All outcome variables were compared using non-parametrical tests (Kruskal-Wallis; Mann Whitney U test for unpaired data). The significance level was set at α = 0.05. A Bonferoni correction was used in case of multiple statistical comparisons.

## Results

### BAL fluid analysis

2-wk, 4-wk and 24-wk CS exposure caused a significant increase in the absolute numbers of total cells, lymphocytes and neutrophils in the BAL fluid (table 2). Significant increase in alveolar macrophages was seen at 4-wk and 24-wk CS exposure.

**Table 2:** Bronchoalveolar analysis

	2-wk Air	2-wk Smoke	4-wk Air	4-wk Smoke	24-wk Air	24-wk Smoke
Total cell number, (× 10^3^)	602.5 ± 41.20	797.53 ± 74.96*	410.00 ± 144.12	1046.00 ± 154.98^†^	432.50 ± 37.97	845.00 ± 114.25^†^
Neutrophils, (× 10^3^)	0.00 ± 0.00	62.59 ± 10.47^‡^	0.00 ± 0.00	200.23 ± 50.97^‡^	0.16 ± 0.16	99.75 ± 30.04^†^
Macrophages, (× 10^3^)	598.55 ± 38.90	723.66 ± 61.59	408.71 ± 142.94	797.55 ± 103.16*	429.46 ± 37.56	719.10 ± 80.27^†^
Lymphocytes, (× 10^3^)	2.49 ± 066	8.32 ± 1.42^†^	1.29 ± 1.21	47.82 ± 8.19^‡^	2.46 ± 0.32	26.15 ± 8.42^†^
Eosinophils, (× 10^3^)	1.46 ± 1.46	2.96 ± 1.49	0.00 ± 0.00	0.39 ± 0.26	0.27 ± 0.18	0.00 ± 0.00

Subacute (4-wk) and chronic (24-wk) CS exposure caused a significant increase in the absolute numbers of total cells, alveolar macrophages, lymphocytes and neutrophils in the BAL fluid, compared to air exposed littermates. (Values are reported as mean ± SEM; n = 8 mice/group, *p < 0.05 versus Air, ^†^p < 0.01 versus Air, ^‡^p < 0.001 versus Air)

### Immunohistochemistry

#### CS induced neutrophilic inflammation in upper airways

**Figure 1:**
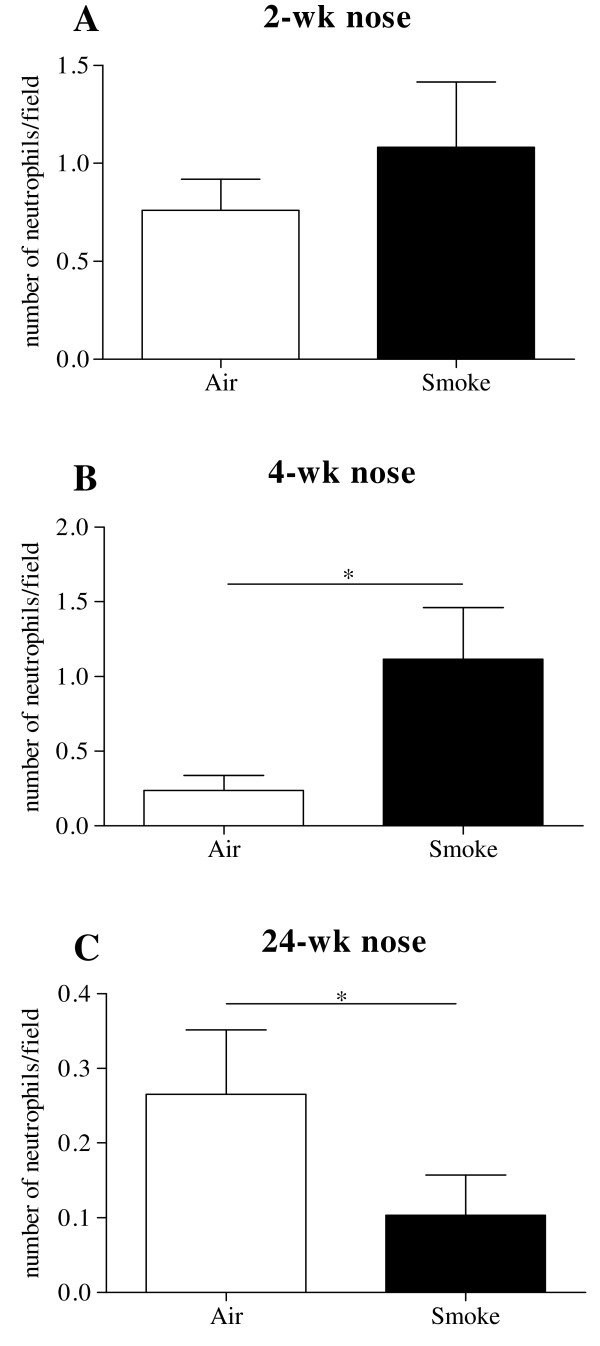
**Average number of neutrophils in nasal turbinate sections**. Increase in number of neutrophils after CS exposure was not seen after 2-wk, compared to air exposed littermates (Fig. 1A), but only after 4-wk (Fig. 1B). Interestingly, the number of neutrophils in the nasal turbinate decreased when the mice were chronically (24-wk) exposed, resulting in a significant lower amount of neutrophils per field in the CS exposed group compared to the air exposed group (Fig. 1C). (n = 8 mice/group, * p < 0.05)

We analyzed the presence of neutrophils in the nasal turbinate tissue of subacute (2-wk and 4-wk) and chronic (24-wk) CS exposed mice by immunohistochemistry, evaluating the average number of neutrophils per high power field, for the entire section. The increase in neutrophils was seen only after 4-wk CS exposure, compared to air exposed littermates (Fig. 1B). Interestingly, the number of neutrophils in the nasal turbinate decreased when the mice were chronically (24-wk) exposed, resulting in a significant lower amount of neutrophils per field in the CS exposed group compared to the air exposed group (Fig. 1C).

#### Scattered CD3+ T cells in nasal turbinates versus (CS-induced) lymphoid follicles in lungs

**Figure 2:**
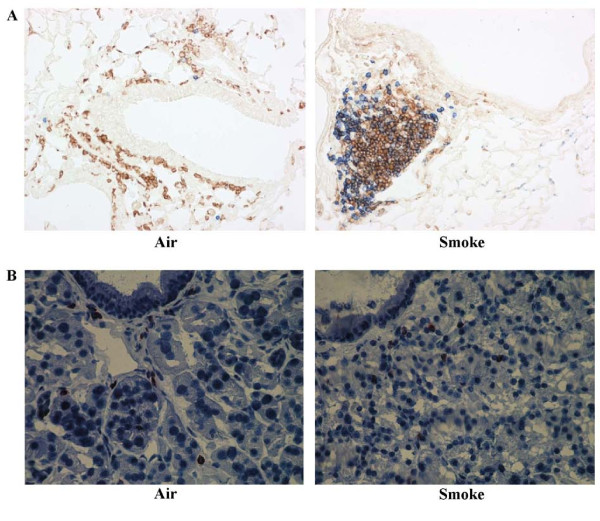
**CD3+ cells**. Lymphoid follicles were demonstrated in lungs after chronic CS exposure, using CD3(brown)/B220(blue) doublestaining (Fig. 2A, × 200). In nose however, no increased number of CD3+ cells in inferior turbinate, or lymphoid follicle neogenesis was found at that time point (Fig. 2B, × 400).

**Figure 3:**
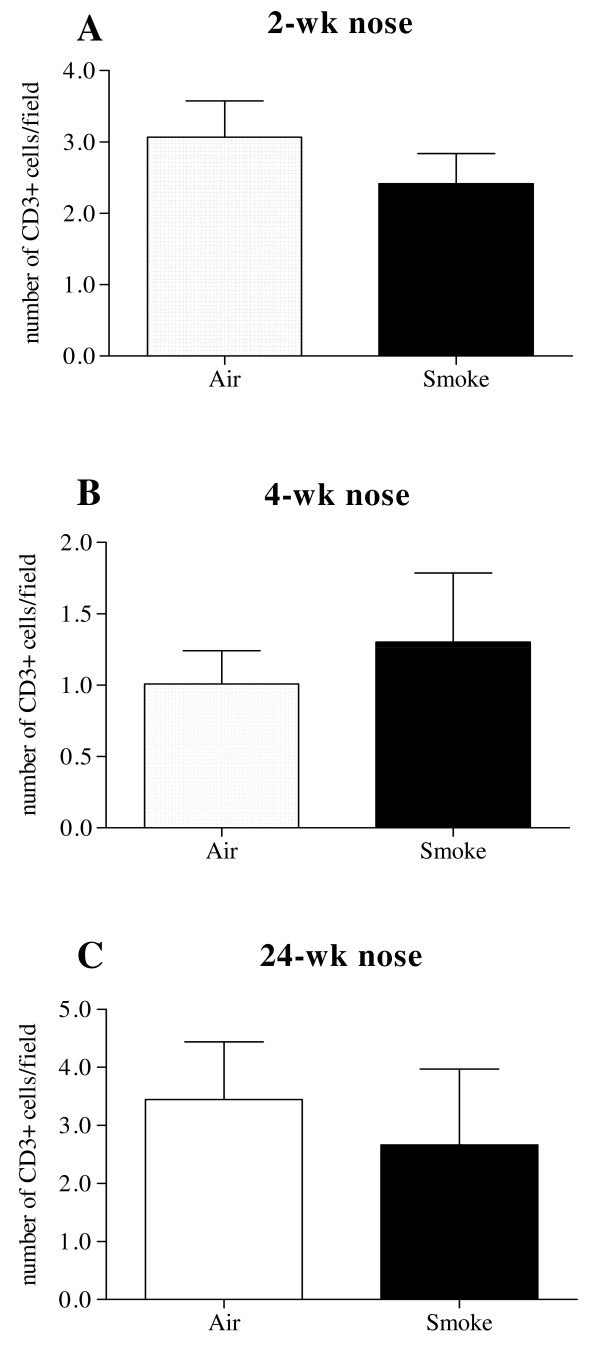
**CD3+ staining**. Nasal turbinate sections were evaluated for the presence of CD3+ cells, within lymphoid follicles. Number of CD3+ cells per field did not differ between air and CS exposed group at any time point (Fig. 3 A-C). (n = 8 mice/group).

The presence of peribronchial lymphoid follicles has been shown both in mice after chronic CS exposure and patients with severe COPD. We could demonstrate the presence of these lymphoid follicles in lungs after chronic CS exposure, using a CD3/B220 double staining (Fig. 2A). Lymphoid aggregates, absent in the bronchovascular lung regions of air-exposed mice, were strongly induced upon chronic CS exposure. In nasal turbinate tissue on the other hand, the number of CD3+ cells did not differ at any time point when air and smoke exposed mice were compared (Fig. 3). Moreover, CD3+ cells were not organized in lymphoid follicles - in contrast to findings in lower airways upon chronic exposure - but were scattered throughout the tissue section (Fig. 2B).

### Real time Quantitative PCR analysis

#### Gene expression analysis in nasal turbinate

##### Neutrophilic chemoattraction related genes

**Figure 4:**
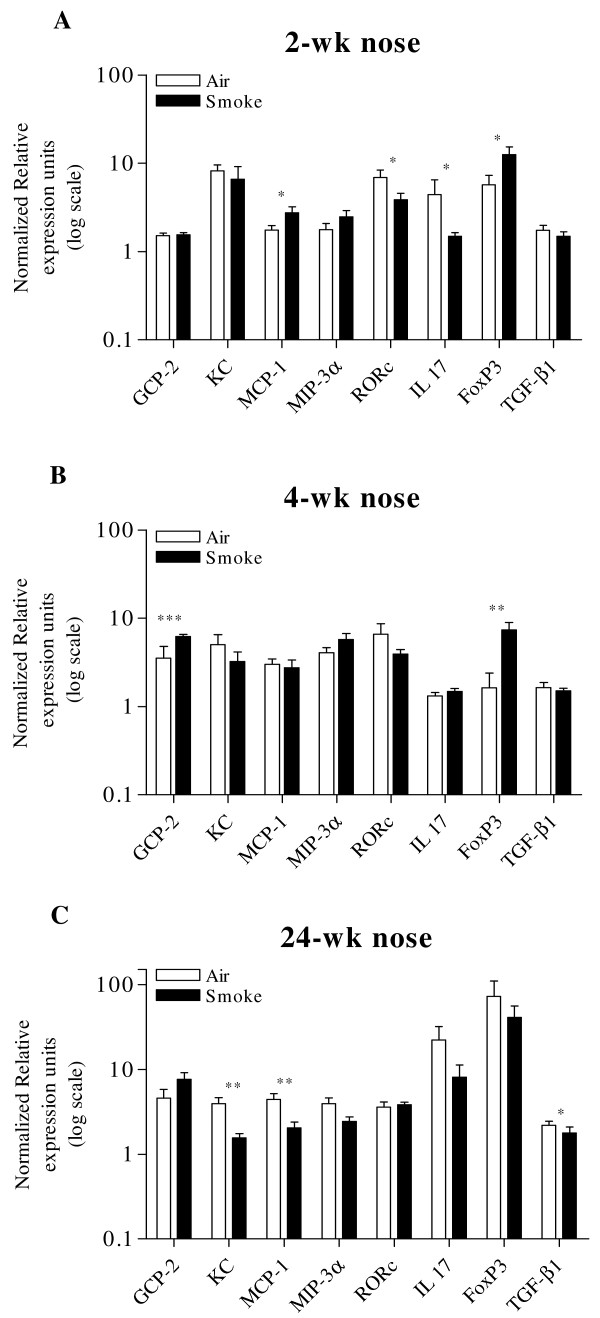
**Gene expression analysis in nasal turbinate**. 2-wk CS exposure resulted in increased levels of MCP-1 and FoxP3. Levels of RORc and subsequent IL-17 were significantly down-regulated at this time point (Fig. 4A). At 4-wk, GCP-2, but not KC, levels are increased. Moreover, FoxP3 is significantly higher in the CS exposed group (Fig. 4B). 24-wk CS exposure results in significant down-regulation of nasal MCP-1, MIP-3α an TGF-β1 (Fig. 4C). (n = 8 mice/group, *p < 0.05, **p < 0.01, ***p < 0.001).

In the nasal turbinates, no significant difference could be found in Granulocyte Chemotactic Protein (GCP)-2 and keratinocyte chemoattractant (KC - mouse IL-8 homologue) levels after 2-wk CS exposure (Fig. 4A). Continued exposure (4-wk) however resulted in significant up-regulation of GCP-2 representing the neutrophilic chemoattractant signal in the CS group compared to the air group, since levels of KC did not differ between groups (Fig. 4B). This increase in GCP-2 expression disappeared at chronic (24-wk) CS exposure; moreover KC levels were significant lower in the CS group at that time point (Fig. 4C).

##### Monocyte/Macrophage chemoattraction related genes

We also found an interesting kinetics in the levels of MCP-1 and MIP-3α. At 2-wk, a significant up-regulation of MCP-1 mRNA in the CS-exposed group and a similar tendency for MIP-3α was seen (p = 0.08, Fig. 4A). This increase disappeared on continued exposure at 4-wk, both for MCP-1 and MIP-3α (Fig. 4B). Moreover, a significant lower expression of MCP-1 and a similar tendency for MIP-3α were noticed at chronic (24-wk) CS exposure (Fig. 4C).

##### T cell related genes

Interestingly, FoxP3 was already significantly increased after 2-wk and 4-wk CS exposure - although this was not the case for TGF-β1 - but not after 24-wk.

Levels of RORc and subsequent IL-17 were significantly down-regulated after 2-wk CS exposure (Fig. 4A), but this finding disappeared when CS exposure was prolonged.

#### Gene expression analysis in lung

##### Neutrophilic chemoattraction related genes

**Figure 5:**
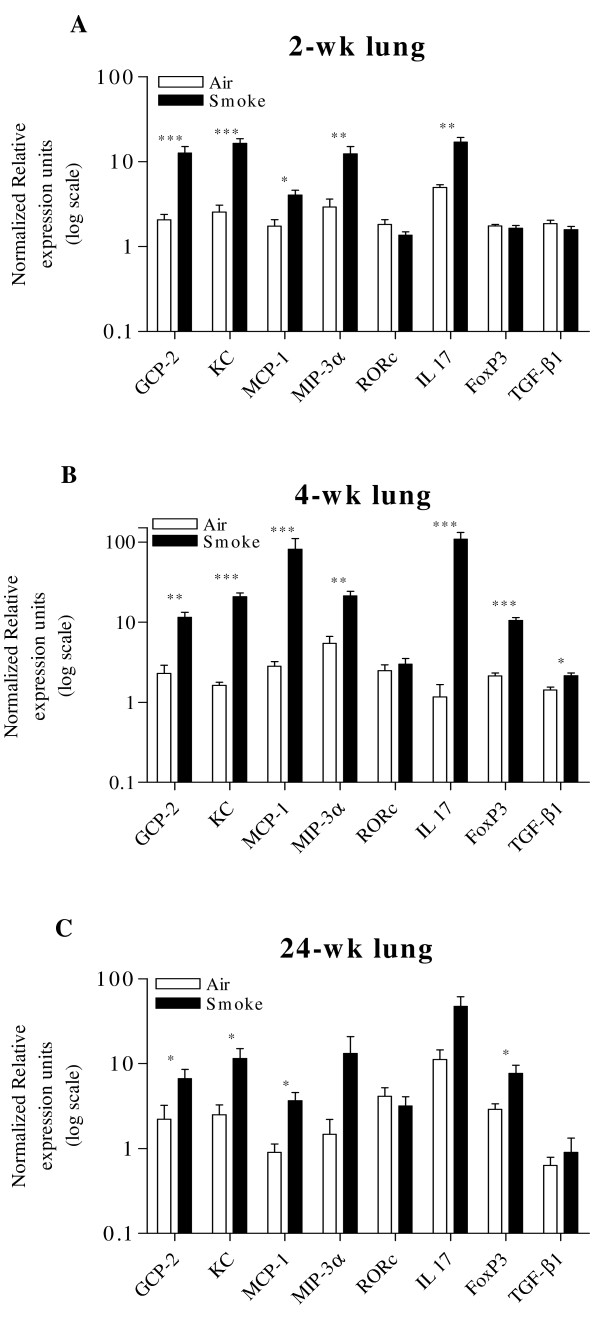
**Gene expression analysis in lung**. Pulmonary levels of GCP-2, KC, MCP-1, MIP-3α and IL-17, but not FoxP3 were significantly increased after 2-wk CS exposure (Fig. 5A). After 4-wk CS exposure, all markers of neutrophilic and monocyte/macrophage chemoattraction are significantly increased, as well as FoxP3 and TGF-β1 (Fig. 5B). Chronic CS exposure caused an increase in levels of GCP-2, KC, MCP-1 and FoxP3 levels (MIP-3α p = 0.05) (Fig. 5C). (n = 8 mice/group, *p < 0.05, **p < 0.01, ***p < 0.001).

Significant up-regulation of both GCP-2 and KC in the CS group remained consistent throughout the entire study, representing the neutrophilic chemoattractant signal triggered by CS exposure (Fig. 5A-C).

##### Monocyte/Macrophage chemoattraction related genes

Both MCP-1 and MIP-3α were significantly increased in the CS group at every time point (except for MIP-3α at 24 wk, p = 0.05) (Fig. 5A-C).

##### T cell related genes

In contrast to the nose, 2-wk CS exposure did not result in increased FoxP3 expression in the lungs (Fig. 5A). At 4-wk and 24-wk however, significantly higher FoxP3 levels were found in the CS exposed groups although we could only find higher TGF-β1 levels at 4-wk (Fig. 5B and 5C).

Although levels of RORc did not differ between experimental groups, IL-17 mRNA levels were significantly increased at 2-wk and 4-wk CS exposure, correlating with the neutrophilic chemoattraction signals.

### Analysis of FoxP3 expression in epithelium vs. subepithelium of nasal turbinates

Recently, FoxP3 expression in epithelial cells has been described [22]. In order to determine the source of FoxP3 expression in whole nasal turbinate, we isolated nasal epithelial cells and subepithelial cells by magnetic cell sorting. The mRNA expression of FoxP3 however was not altered in the nasal epithelium after 4-wk CS exposure (Air 0.3453 ± 0.0084 versus Smoke 0.2894 ± 0.0084 normalized relative expression units). On the contrary, we demonstrated a nearly 5-fold increase in subepithelial FoxP3 expression in nasal turbinates upon 4-wk CS exposure, possibly due to infiltrating T regulatory cells (Air 1.0432 ± 0.0723 versus Smoke 5.1730 ± 0.9323).

## Discussion

In this study we aimed to investigate the effects of cigarette smoke (CS) on upper airways and lower airways, in a mouse model of subacute and chronic CS exposure. We here demonstrate for the first time that the inflammatory response upon CS exposure clearly differs between nose and lungs in mice. The nature and kinetics of both the neutrophil and monocyte/macrophage inflammation differ in both airways compartments. This indicates the involvement of different regulatory mechanisms, which is reflected by the observed differences in FoxP3 increase after CS exposure. The suppressive mechanisms arise earlier and appear to be more efficient in nose than in lungs. Although increased levels of MCP-1, MIP-3α and GCP-2 are found both in nose and lungs after subacute CS exposure, the neutrophilic influx and increase in neutrophilic chemoattraction signals are transient in upper airways while they remain constant in lower airways. Consequently, chronic upper airway CS exposure results in a non-inflammatory status with a significant downregulation of inflammation, while lower airway inflammation is clearly present and ongoing.

Neutrophilic inflammation in the nasal turbinate tissue was not present after 2-wk CS exposure, likely due to the absence of a neutrophilic chemoattraction signal, as both GCP-2 and KC levels were not increased in the CS group. However, prolonged (4-wk) exposure caused a significant GCP-2 increase in the CS group, which correlates with the immunohistochemistry, showing a higher number of neutrophils per field in the CS group compared to the air group, but only after 4-wk. To our surprise, chronic (24-wk) CS exposure did not cause a further increase in neutrophil accumulation in the nasal turbinate tissue. Moreover, GCP-2 levels and KC levels in the CS group did not differ and were significantly down regulated from controls respectively. This was again confirmed by IHC, where we found a significant decrease in the number of neutrophils per field in the CS group compared to controls. This may be interpreted as a clear sign of down-regulation of the neutrophilic inflammatory long-term response in the nasal turbinates. Evaluation of neutrophilic inflammation in upper airways was done in nasal turbinate tissue, because nasal lavage did not yield sufficient cells allowing a reliable cell differentiation. As a consequence, compartmentalization of inflammation in both upper and lower airways may influence the interpretation of these findings. Indeed, cigarette smoke causes an increase of neutrophil numbers in BAL (mouse studies), or sputum (human studies), whereas its effect in lung tissue or biopsies is less pronounced.

Our findings on neutrophilic inflammation in upper airways are in sharp contrast with the data obtained from experiments in the lung, where CS exposure resulted in a significant increase in both GCP-2 and KC at all time points, accounting for to the observed influx of neutrophils in the BAL fluid of these mice [23].

We have shown a remarkable change over time in the nasal mRNA MCP-1 levels of CS exposed mice, showing an initial increase, followed by a significant decrease in MCP-1 levels in the nasal turbinate upon chronic exposure. In the lungs of these mice however, we detected a consistent increase in MCP-1 levels in CS exposed mice on each time point [23]. This is another sign of the different inflammatory response to CS in the upper airway.

The role of pro-inflammatory T helper 17 phenotype in the pathogenesis of COPD is increasingly studied, and it is suggested that COPD might be better explained by the Th17 phenotype [12]. These Th17 cells, which require the up-regulation of the orphan nuclear receptor RORgammat (encoded by RORc) for differentiation from naïve T cells [24], account for the production of several members of the IL-17 family of cytokines, which have proven abilities to recruit and activate neutrophils [25]. Here, nasal mRNA levels of RORc and IL-17 in the nose were significantly down-regulated after 2-wk CS exposure, but not upon longer (4-wk and 24-wk) exposure. In lungs however, the response of Th17 cells appears to be opposite, as 2-wk and 4-wk CS exposure resulted in a significant up-regulation of IL-17, and chronic (24-wk) exposure showed a similar tendency. These differences in IL-17 levels between nose and lungs, can explain the observed differences in neutrophil accumulation, as described above.

T regulatory cells expressing FoxP3 are thought to play a role in controlling CS induced inflammation [15,26], amongst others via the immunomodulatory cytokine TGF-β1 [27]. In nose, FoxP3 mRNA expression was increased already after 2-wk, and was mainly found - at least at 4-wk - in the subepithelium, possibly due to invading Tregs expressing FoxP3. In lungs, FoxP3 was only increased after 4-wk, which is in line with increased Tregs in lungs after CS exposure [14]. Interestingly, these infiltrating Tregs in lungs are thought to have a weak functionality, as they are unable to control inflammation in lungs [15]. It is tempting to speculate that Tregs act early and adequately in nose to suppress CS-induced inflammation, but that they invade later and have weaker functionality in lungs, allowing inflammation to persist. Alternatively, the CS exposure of the nose might be higher in mice - obligatory nose-breathing animals - compared to lungs, allowing tolerazation or change in cell populations to occur earlier. Indeed, upon 24-wk CS exposure the number of neutrophils shows a decreasing tendency compared to 4-wk CS exposed mice.

Although *in vivo *cigarette smoke-exposed mice can offer valuable information on several aspects of the pathogenesis of COPD, such as the time course of upper and lower airway inflammation, there are also limitations that need to be taken into account. Firstly, a number of anatomical and physiological differences exist between the respiratory tract of mice and humans. For example, mice are obligate nose breathers that filter tobacco smoke inefficiently, and they have less branching of the bronchial tree. Furthermore, the profile of inflammatory mediators is also slightly different in the mouse. And lastly, there is no mouse model that mimics all the hallmarks of COPD pathology, including exacerbations and extrathoracic manifestations.

Another possible limitation to this study is the fact that not only T cells are able to produce either IL-17, TGF-β or FoxP3, but a number of other cells like neutrophils or epithelial cells can do so. Furthermore, the suppressive capacity of the FoxP3 producing Tregs in upper airways stills remains to be elucidated.

Although the inflammatory answer of nose and lungs is clearly different upon CS exposure, possible confounding factors might influence the data interpretation in this model. Above, we have described the issue of compartmentalization of inflammation, and the relative dosage exposure, with higher deposition of CS in the nose vs. lungs. Furthermore, physiologic temporal changes are seen in the inflammatory readouts: levels of inflammatory cells and mediators of unexposed control mice vary over time, as shown in Fig. 3, 4, 5. By using age-matched control mice in our experiments, we have corrected for these physiologic temporal changes. Altogether, the above mentioned limitations of this model remain to be elucidated.

## Conclusions

In conclusion, we have demonstrated that cigarette smoke induced inflammation differs between nose and lungs in this mouse model. After CS exposure, inflammatory markers were upregulated in lungs at all time points. However, this was not the case in the nose, where particularly upon chronic CS exposure, nasal inflammatory markers were significantly lower than the control (air) conditions. It is possible that infiltrating FoxP3 expressing Tregs might account for these observed differences, although further investigation is necessary to identify possible differences in their suppressive functionality in both airway compartments.

## List of abbreviations

ACTB: beta-actin; GCP-2 (CXCL6): granulocyte chemotactic protein 2; KC (CXCL1): keratinocyte chemoattractant; MCP-1 (CCL-2): monocyte chemotactic protein-1; MIP-3α (CCL-20): Macrophage Inflammatory Protein-3 alpha; RORc: orphan nuclear receptor RORgammat; IL-17: Interleukin 17; FoxP3: Forkhead box P3; TGF-β1: Transforming growth factor beta 1

## Competing interests

The authors declare that they have no competing interests.

## Authors' contributions

WH carried out the design and coordination of the study, gathered the data on upper and lower airway inflammation, interpreted the data, drafted and finalized the manuscript. CP-N developed and optimized the PCRs on nose and lung samples. LD designed and optimized the nasal epithelial cell isolation procedure. OK optimized and carried out the IHC staining of the nasal turbinates. TM and KB were involved in the coordination and design of the study, and the critical reading of the manuscript. NP and LR provided mice and were involved in the experimental design of the CS-induced airway inflammation. GJ, GB and CB participated in the coordination of the study, helped to interpret the data and critically revised the manuscript. All authors read and approved the final version of the manuscript.
